# Elements and roadmap for interactive molecular graphics and modeling “in the Holodeck”

**DOI:** 10.1002/pro.70457

**Published:** 2026-01-20

**Authors:** Adrian J. Mulholland, Luciano A. Abriata

**Affiliations:** ^1^ Centre for Computational Chemistry, School of Chemistry University of Bristol Bristol UK; ^2^ Institute of Bioengineering School of Life Sciences, École Polytechnique Fédérale de Lausanne (EPFL) Lausanne Switzerland

**Keywords:** AI agents, artificial intelligence, augmented reality (AR), collaboration, extended reality (XR), human‐computer interaction, interactive molecular dynamics, large language models, mixed reality, molecular graphics, molecular manipulation, molecular modeling, multi‐modal interaction, protein science, structural biology, virtual reality (VR), WebXR

## Abstract

Molecular graphics have been instrumental in advancing chemistry, drug discovery, materials science and structural biology, enabling visualization of molecular systems from static images to dynamic web displays and immersive platforms. While effective visualization is largely a solved problem, in this perspective we argue that the next significant leap lies beyond passive viewing. Rather, the frontier is in enabling intuitive, immersive, direct 3D manipulation and interaction with molecular systems to address the inherent limitations of carrying out complex 3D tasks via 2D interfaces. The vision, which is starting to be realized, is of immersive molecular science environments in which researchers leverage multi‐modal inputs that feel natural (with hands plus possibly haptic or pseudo‐haptic feedback, voice user interfaces and AI‐based assistance), collaborating seamlessly in concurrent sessions, naturally visualizing and connecting different types of data and models, and engaging with molecules assisted by real‐time simulation engines. Many of the technologies required to develop such environments already exist, at least in basic forms. In this Perspective, we discuss current prototypes and software solutions that incorporate some of the elements needed, and that are available for use. We discuss applications and practical demonstrations, and outline the developments that are required to make the Molecular Holodeck a reality. We also discuss challenges that need to be addressed in order to achieve this vision. The coming shift toward hands‐on, multiuser, immersive, natural and physics‐informed manipulation will transform hypothesis generation, molecular design, fundamental understanding, collaborative working, discussions, and thus research and education in the chemical sciences, as others have envisioned for “molecular visualization in the holodeck.”

## 1 INTRODUCTION

**FIGURE 1:**
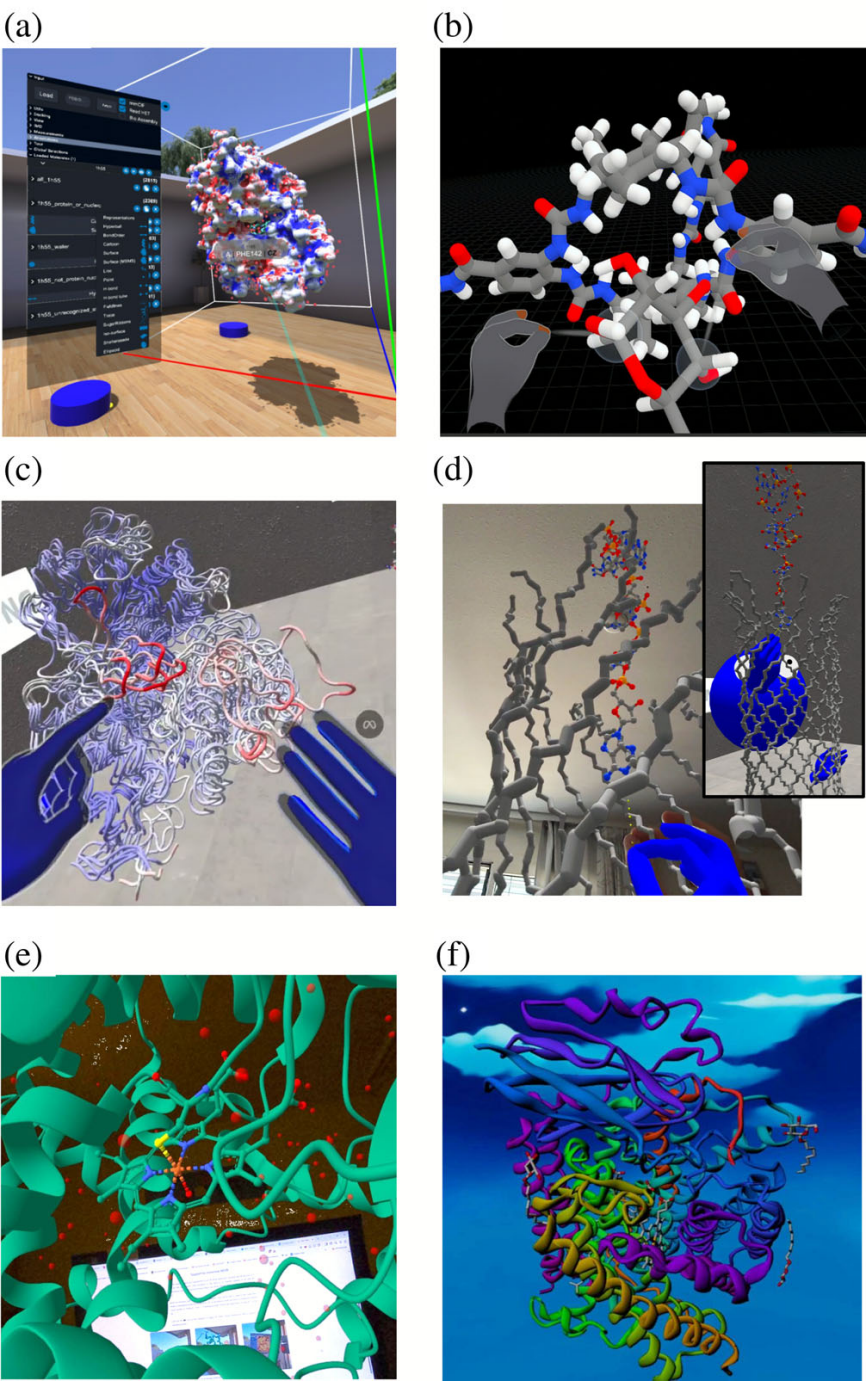
Screenshots exemplifying software for immersive molecular visualization running in head‐mounted displays as of 2025. (a) UnityMol (https://unity.mol3d.tech/) displaying horseradish peroxidase with its surface colored by electrostatic potential and with information about the atom being pointed at. Notice the rich set of features and commands available in this program. (b) Nanover (https://nanover.org/) running an interactive MD simulation in explicit solvent (water molecules hidden), in which a user interacts with the molecules by pulling atoms to achieve a specific task. (c) A MolecularWebXR (https://molecularwebxr.org/) session in VR, in which a teacher is explaining about a flexible loop in malate G synthase. This uses the Structural Biology Course room to be released in the next website update. (d) The forthcoming MolecularWebXR‐integrated HandMol (to become available inside MolecularWebXR.org in its next update) in AR, where a user is pulling a piece of single‐strand DNA into a protein nanopore for subsequent export and downstream work. The main panel shows the user's own view in augmented reality, while the inset shows the user from the side as seen by a second user accessing from a laptop. (e) The web molecular viewer Mol* recently added WebXR functionalities too; here, it is shown running in AR. (f) ProteinVR, one of the earliest Web‐based systems for immersive molecular graphics. (a)–(f) All these screenshots were taken from inside the AR/VR headsets, but the immersive views in tools like MolecularWebXR, HandMol, Mol*, ProteinVR and VRmol can all be used out of the box in regular smartphones and tablets too, thanks to their WebXR core. We thank Dr. S. A. Siddiqui, K. Yang and Dr. M. Hanzevacki from the Mulholland lab for providing the screenshots shown in panels (a) and (b).

Our ability to understand the architectures of molecular systems has been fundamentally shaped by computational molecular graphics, which today drive research and education in modern structural biology, chemistry, materials sciences, and other molecular sciences (Richards, 2022). From the foundational clarity of illustrations like those of David Goodsell, whom this special issue honors, to the dynamic interactivity of desktop software (PyMOL, ChimeraX, VMD) and web apps and libraries (JSmol, NGL Viewer, Mol*), molecular visualization has become ubiquitous and powerful. Immersive technologies add another layer that goes into 3D (as opposed to the 2D‐based, in most current molecular graphics software) representations of the inherently 3D molecular systems, and (unlike, e.g., stereoviews or 3D displays) immerse the user in the 3D molecular environment. Immersive visualization enhances the sense of scale and presence, which can be particularly effective for collaborative exploration and for educational purposes in chemistry and biology; its effectiveness (and superiority to traditional 2D methods for some applications) has been demonstrated through rigorous user tests (O'Connor et al., 2018; Rodríguez, Frattini, et al., 2021). Often referred to under the umbrella term extended reality (XR), immersive visualization includes virtual reality (VR), which provides a fully digital experience separate from the physical world, augmented reality (AR), which overlays digital information onto the real‐world environment, and mixed reality (MR), which blends the real and virtual, allowing digital objects to interact with the physical space. The potential of XR in molecular and material research has been recognized for many years, and is now starting to be realized as technological developments (hardware and software) make such methods accessible and practically useful. XR software for molecular graphics has been available for some time, such as the feature‐rich UnityMol (Doutreligne et al., 2014) and Nanome, the web‐based ProteinVR (Cassidy et al., 2020), VRmol (Xu et al., 2021), MoleculARweb (Rodríguez et al., 2023; Rodríguez, Frattini, et al., 2021; Rodríguez, Krapp, et al., 2022), MolecularWebXR (Cortés Rodríguez et al., 2025), the HandMol prototypes (Abriata, 2025; Rodriguez et al., 2023; Rodriguez et al., 2025) and Mol*'s latest version (Sehnal et al., 2021), the interactive MD‐enabling Narupa and NanoVer (Jamieson‐Binnie et al., 2020; Stroud et al., 2025), and the VR extensions of ChimeraX and VMD (example screenshots in Figure 1). Various comprehensive reviews cover these tools as well as more general aspects of molecular visualization in immersive environments (Baaden & Glowacki, 2025; Fombona‐Pascual et al., 2022; Kozlíková et al., 2017; Kuťák et al., 2023), elaborated upon by exciting perspectives by D. Goodsell (O'Donoghue, Gavin, et al., 2010; O'Donoghue, Goodsell, et al., 2010; Richardson et al., 2021) among others (Kozlíková et al., 2017; Kuťák et al., 2023; Martinez et al., 2019, 2020), all motivators of our own work, together with T. Goddard's view of molecular graphics happening “on the holodeck”, referring to the ultrarealistic immersive simulation systems portrayed in the Star Trek series (Goddard et al., 2018). In our view, this vision of a “Molecular Holodeck” extends the concept to a globally connected space, in which collaborators can meet and interact with shared molecular systems regardless of their physical location; that is, they can meet both physically and virtually, as in the TV series’ Holodeck, or remotely, or mixing both presential and online concurrency.

## 2 INTERACTING WITH VIRTUAL MOLECULAR SYSTEMS IN IMMERSIVE 3D

Despite the advancements and superior experiences offered by immersive graphics, a fundamental challenge still remains: actively interacting with and modifying 3D structures relies largely on indirect, learned conventions mediated by 2D input devices. While visualization software helps us to see molecules and materials, the process of doing—manipulating, building, designing—is still largely constrained by the interface. In fact, one could argue that conventional 2D interfaces are already remarkably effective at visualization in its strict meaning: mechanisms for stereoscopic view, depth cueing, intuitive rotation/zoom controls, and standardized representations solve many 3D perception problems quite adequately, at least after some training. It is hence not immediately clear whether enhanced passive immersive viewing can fundamentally change scientific work and education, that is, whether it can lead to discoveries that were previously inaccessible or it can allow students to learn faster and/or better; however, some very complex scenes become much clearer when visualized immersively, for example, electric fields assisting enzyme catalysis (Suardíaz et al., 2025). Some aspects of structure (e.g., stereochemistry and dynamics) and properties (e.g., vector fields) are certainly more easily perceived in 3D. While undoubtedly valuable to grab attention in educational and outreach settings and useful in some cases such as that just mentioned, and perhaps as a curiosity in certain kinds of scientific presentations, the argument that immersive visualization is essential remains debatable, especially considering the practical hurdles of cost, specialized hardware, potential discomfort (like VR sickness), and resolution limitations compared to the convenience and fidelity of standard monitors. However, we argue that the case is completely different for molecule manipulation in 3D space.

For molecule manipulation, 2D‐based inputs are inefficient, tedious, even physically uncomfortable, and largely single‐user, which hampers efficient collaboration. 3D immersive technologies can make a transformative difference here, promising a genuine revolution by enabling natural 3D interaction of users with molecular systems and with each other. The next major frontier lies beyond visualization, in the realm of intuitive, direct manipulation. Manipulating 3D objects using an inherently 2D input device (such as a computer mouse) requires learning abstract mappings (e.g., click‐and‐drag combinations with left, center, or mouse button, plus key pressing in some cases). While powerful once mastered, these mappings are often unintuitive, especially when it comes to rotating, translating, or modifying parts of the molecular systems. Besides, prolonged action on a mouse might cause muscle stress. Immersive systems, equipped with tracked controllers or with hand‐tracking capabilities, allow for direct, physical‐like interaction with virtual molecular models; this is where we propose that transformative potential lies, together with the possibility for multiple users to simultaneously act in the same session. Thus, the true advantage of immersive technologies is not just to change our viewpoint from 2D to 3D, but rather to fundamentally alter our ability to interact with molecular data and with other users, as others have also recognized and have begun to address (Deeks, Walters, Hare, et al., 2020; Walters et al., 2022a).

Imagine physically grabbing a ligand molecule and intuitively maneuvering it into a protein's binding pocket, perhaps even receiving haptic feedback for steric clashes. Or bending a flexible protein loop with your hands to explore conformational possibilities, or to sculpt it into a cryo‐EM density map—tasks for which specialized immersive tools for crystallographic model building are already being developed and validated (Meng et al., 2023; Todd & Emsley, 2021). All this not alone, but together with colleagues, as you all discuss the system together. Likewise, consider a teacher showing how two molecules interact or change conformation, perhaps even the student probing the interactions hands‐on like with plastic molecular models but augmented with more realistic calculations of molecular mechanics.

A compelling vision for this blend of interaction and physical realism is demonstrated by interactive molecular dynamics (iMD) systems, such as MolPlay (Baaden, 2024) and, for immersive iMD, the Narupa and NanoVer frameworks developed by Glowacki, Mulholland, and colleagues (Baaden & Glowacki, 2025; Bennie et al., 2019; Deeks, Walters, Hare, et al., 2020; Jamieson‐Binnie et al., 2020; O'Connor et al., 2018, 2019; Stroud et al., 2025; Walters et al., 2022b). These platforms allow users in VR to directly apply forces onto atoms of a molecular simulation and witness the consequences of their actions in real time, effectively “feeling” the energy landscape—which apparently humans can perceive even without haptic controls, through appropriate visual cues (Roebuck Williams et al., 2024; Williams et al., 2020). A variety of methods can be used as the simulation engines: empirical “molecular mechanics” forcefields allow for realistic modeling of the structure, dynamics and interactions of macromolecules and materials, while approximate quantum mechanics methods, or increasingly machine learned potentials, allow for interactive modeling of the bond breaking and making in chemical reactions. Such tools are not only powerful for research, for example in studying ligand binding or reaction pathways, setting up systems for simulations or probing reactivity (Baaden & Glowacki, 2025; Bennie et al., 2019; Deeks et al., 2023; Deeks, Walters, Hare, et al., 2020; Walters et al., 2022a), but also hold immense potential for education, allowing students to develop an intuitive grasp of molecular behavior by actively participating in simulated events rather than passively observing them.

While the field would undoubtedly benefit from more standardized, cross‐platform benchmarks, a significant and growing body of evidence already demonstrates the tangible advantages of immersive interaction for specific, complex scientific tasks. User tests have shown that for some molecular modeling problems, interactive VR is superior to traditional mouse and screen interfaces. O'Connor et al. rigorously demonstrated benefits of interactive VR, showing it to be superior to standard mouse/screen interfaces for some molecular modeling tasks (O'Connor et al., 2018). Interactive MD in VR has also been successfully applied to challenging drug discovery tasks, such as flexible protein‐ligand docking, where tests showed that even novices in VR were able to reproduce crystallographic binding poses with good accuracy (Deeks, Walters, Hare, et al., 2020). The same Interactive VR‐MD approach was used to create models of SARS‐CoV‐2 main protease with peptide substrates; protocols for this were tested, and results shown to be useful by comparison with previous experiments on the original SARS‐CoV Mpro (Deeks, Walters, Barnoud, et al., 2020).

Beyond discovery, immersive environments have shown great potential for education and engagement. Students reported improved educational outcomes and interest when learning about enzyme catalysis in VR compared to traditional methods. Moreover, in a survey following the application of interactive VR to education on enzyme structure and mechanisms, the students indicated that it improved their perceived educational outcomes and their interest, finding iMD‐VR more engaging than traditional screen‐based computational chemistry approaches (Bennie et al., 2019).

In turn, this potential for engagement has been extended to the “gamification” of chemical discovery, where users in a game‐like environment can drive and map out complex molecular transformations such as chemical reaction pathways by manually guiding simulations with knowledge and intuition. In turn, this allows identification and machine learning of the complex collective variables/reaction coordinates that describe such processes, for example, for use in subsequent enhanced sampling simulations. This has been demonstrated, for example, in the calculation of free energy profiles for chemical reactions between propyne and hydroxyl radicals, and for the dissociation of benzamidine from trypsin (Deeks et al., 2023; Shannon et al., 2021). In the area of materials for heterogeneous catalysis, interactively guiding the diffusion of small molecule promoters through a zeolite led to the identification of bottlenecks restricting diffusion and a specific “three‐point turn” motion (Crossley‐Lewis et al., 2023) which subsequent molecular dynamics simulations verified and further dissected (Dunn et al., 2024)—an example of how human intuition in interactive VR produced a discovery, then further studied with conventional methods.

These examples show that the potential of interactive VR is now being realized in molecular and materials science. Interactive VR will certainly facilitate and accelerate discovery in biomolecular science, materials, and catalysis. In structural biology, it provides not only for visualization of molecular structures but also density of, for example, assemblies, organelles, and virions from tomography and electron microscopy. Inspired by the art of David Goodsell, one can envisage “zooming in” from the level of a cell to dynamic, molecular level models of cellular components and processes; and “zooming out” to visualize larger scales, switching between levels of resolution/detail, and combining data from different experimental techniques and resolutions in interactive multiscale models (Amaro & Mulholland, 2018). The challenge ahead is to build on these compelling individual successes and move toward establishing standardized benchmarks for representative tasks to systematically quantify where and how much of a measurable advantage the “Molecular Holodeck” can provide.

## 3 BEYOND IMMERSIVE MANIPULATION

Intuitive manipulation extends far beyond just controlling the molecular systems via natural moves in 3D space. Future interactive environments should embrace multi‐modal interaction to create richer and more efficient workflows. Direct hand manipulation could be coupled with increasingly sophisticated speech recognition, potentially leveraging large language models (LLMs) for natural language understanding. A researcher could verbally instruct the system to “freeze the backbone atoms,” “show hydrogen bonds to the ligand,” or “run a quick minimization on this sidechain” while simultaneously performing fine‐grained adjustments by hand. Importantly, such voice user interface (VUI, as opposed to GUI) could allow for complex operations without cumbersome menus or context switching, with no need to step outside the immersive environment. Complementing such developments, multi‐sensory feedback, including auditory cues synthesized in real‐time, could confirm actions, report calculated energies, or signal steric clashes, creating more comprehensive understanding than visuals alone can provide.

The HandMol protypes (Rodriguez et al., 2023), for instance, provide a range of proof‐of‐concept and feasibility tests exploring these very ideas, integrating preliminary versions of hand tracking, voice control assisted by LLMs (Abriata, 2022), and auditory feedback within a WebXR framework, with some other elements described below. While promising and inspiring as a demonstration of what is possible with current technology (available for use with any WebXR‐enabled, Rodríguez, Dal Peraro, & Abriata, 2021; Abriata, 2017, 2020, headset), these prototypes underscore that significant development is still needed to create robust, seamlessly integrated systems ready for widespread scientific application.

## 4 MULTIUSER (HUMAN–HUMAN) COLLABORATIONS

**FIGURE 2:**
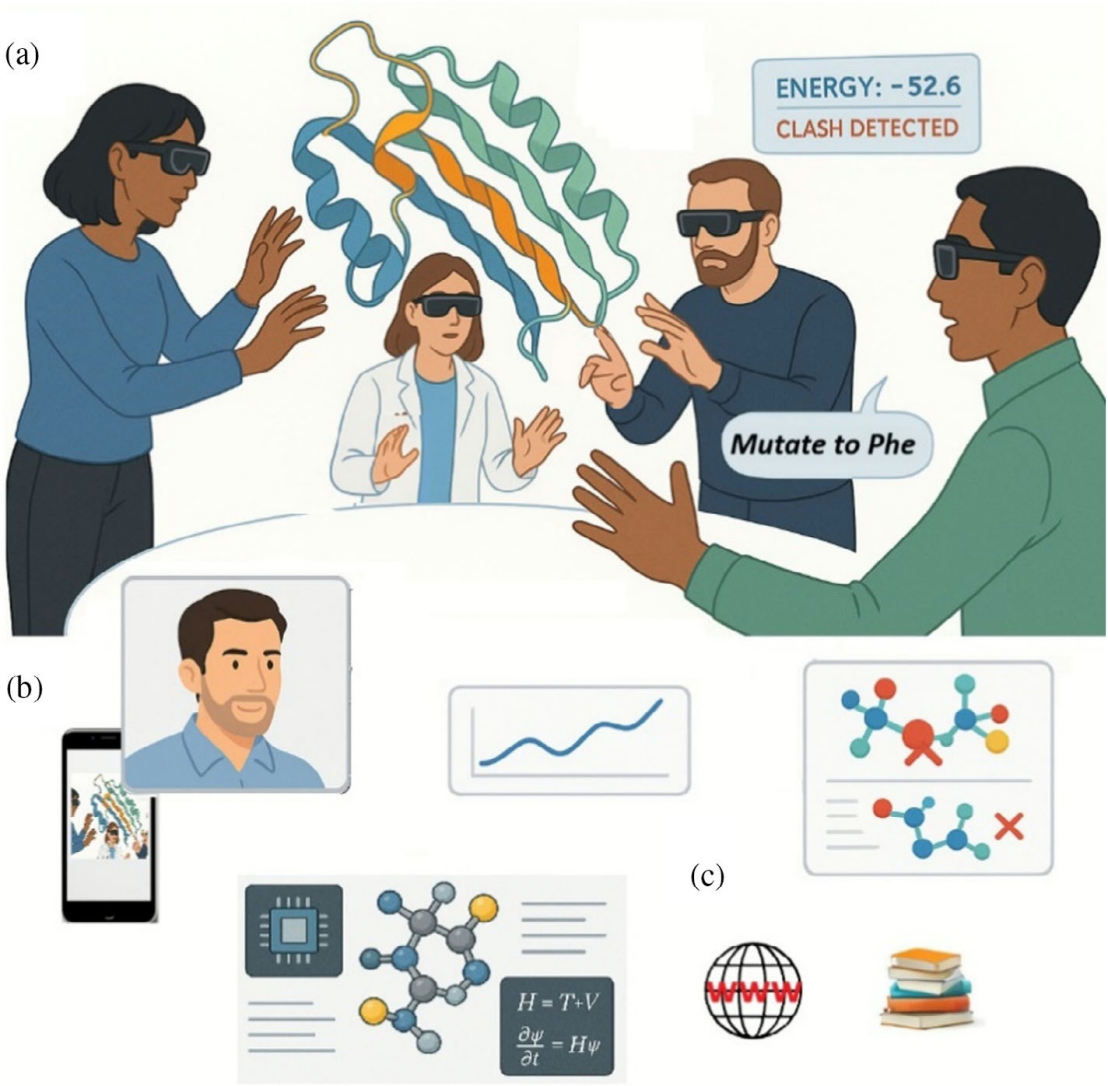
A vision of the “Molecular Holodeck” for scientific discovery and education in immersive, dynamic, collaborative environments where intuition and natural interaction thrive. (a) Researchers (or students and educators, etc.) meet in a shared immersive space to directly manipulate molecular structures with their hands and assisted by computer systems. (b) Users can also join remotely with AR/VR headsets, or possibly even via devices not specialized for AR/VR such as phones, tablets, or laptops. (c) The environment acts as an intelligent partner, responding to natural language voice commands via an AI assistant and providing real‐time feedback from realistic simulations and estimations of experimental observables, as well as from literature and database consultations. Picture composed from ChatGPT and Krea. AI generations plus manual interventions.

Scientific discovery is rarely a solitary pursuit; thus, the interactive environments of the future must be fundamentally collaborative. These platforms should seamlessly support two primary modes: remote collaboration, where geographically dispersed users meet in a shared virtual space, and co‐located collaboration, where multiple users in the same physical room interact with shared mixed‐reality models. The co‐located experience is made possible by modern headset technology, which uses environment scanning and spatial anchors to build a common coordinate system that all devices can share (driven by consumer applications and demand, high‐quality headsets with these capabilities are increasingly accessible that require no specialist equipment and are easy to use). Naturally, these modes are not mutually exclusive and can be combined into hybrid sessions that involve local and remote participants to interact simultaneously, as depicted in Figure 2. The practical power of remote collaboration has been demonstrated in applications relevant to drug discovery: during the COVID‐19 pandemic, researchers in different physical locations worked together in VR in designing novel peptide inhibitors for the SARS‐CoV‐2 main protease, modeling their binding to the enzyme in a collaborative project with experimental validation (Shannon et al., 2021).

Today, tools such as Nanome and MolecularWebXR allow multiuser visualization, and some (e.g., Narupa and NanoVer) even allow multiple users to engage in interactive simulations. In the HandMol prototype, WebRTC enables direct peer‐to‐peer communication between either a headset and a computer or two headsets—without even requiring a server, other than for the first link. With such a setup, users can easily move structure files in and out of the headset, allowing them to start a modeling session, modify the molecular system by hand, perhaps assisted by a forcefield, and then save the edited coordinates directly to the computer for downstream work. Meanwhile, the setup using two headsets allows two users to meet in a joint immersive session, see each other's hands, and collaborate on a molecular system naturally and regardless of their physical locations.

The MoleculARwebXR web platform and associated tools (Cortés Rodríguez et al., 2025; Rodríguez, Dal Peraro, & Abriata, 2021, 2022) take this to a higher level by centralizing the virtual scenes in a server where not only two, but at least as many as 8 users can connect together (Cortés Rodríguez et al., 2025). While this platform only allows the display and handling of virtual objects that behave as a whole, we are at advanced stages of a setup that combines HandMol‐like molecular visualization and manipulation in XR with MolecularWebXR‐like multiuser capabilities, to create a website where users can inhabit the same space to simultaneously view and manipulate the same molecular system by interactively acting on a simulation, all at the same time and each from their preferred point of view. We described this upcoming MolecularWebXR‐integrated HandMol in a recent preprint (Abriata, 2025).

An important consideration is that unconstrained manipulation of molecules risks creating physically unrealistic scenarios. To avoid this, the interactive systems must integrate manipulation with some kind of physical and/or chemical calculations. This does not necessarily require the full computational cost of continuous iMD, although such frameworks represent a powerful paradigm for “feeling” energy landscapes directly. For example, the external computational engines could simply run minimizations or short equilibrations only on demand, as in the HandMol prototype, which runs rapid energy minimizations using an AMBER force field or the DFT‐trained ANI neural network potential (Devereux et al., 2020; Gao et al., 2020; Smith et al., 2017) every time the atoms are released by the users or upon request. This approach allows researchers to intuitively sculpt or probe molecular behavior while remaining grounded in physical reality, using computation not just for post‐hoc analysis but as an integral part of the interactive exploration process. Meanwhile, in an upcoming merge of HandMol and MolecularWebXR, the users interact with simulations as they run on‐the‐fly in an MD engine, in immersive XR, like in the pioneering systems Narupa and NanoVer, but available off‐the‐shelf and in any browser‐enabled device on the web.

## 5 HUMAN–COMPUTER COLLABORATION

Beyond human‐human collaboration and seamless molecular manipulation, the ultimate “Holodeck” experience for molecular graphics and modeling would involve highly natural and smooth human‐computer partnerships. The immersive environment should not stand as a mere passive stage, but rather serve as an active assistant. Real‐time simulation of molecular mechanics, providing instantaneous feedback on the energetic consequences of user manipulations, simulating experimental observables such as SAXS profiles or chemical shift perturbations in real time, are example elements of this as covered above. However, there are other ways in which computers can assist human users as they work or study, as we describe next.

LLMs, and particularly agents and so‐called “reasoning models,” could serve as intelligent assistants within immersive workflows: they could not only help to interpret complex verbal commands at the core of VUIs, but also anticipate user needs by suggesting relevant operations or representations, or even help troubleshoot problematic structures by identifying unlikely geometries, missing atoms, strained interactions, and so forth. Furthermore, LLMs could act as powerful data and literature retrieval agents, rapidly fetching and displaying molecules and other data related to those being studied, showing relevant information such as known effects of mutations around an inspected region, or recognizing relevant chemical moieties (such as, for example, enzyme active sites, etc.), all without leaving the immersive environment. Such smart aid could dramatically accelerate the research cycle by seamlessly integrating knowledge discovery with interactive modeling, creating a truly synergistic environment where the user's intuition is augmented by computational power and data to model the underlying mechanics and its instant access to a vast knowledge base that it can also process and “understand.” Such a system could even learn from expert user interactions over time, perhaps through imitation learning strategies, to provide increasingly sophisticated and context‐aware assistance. Of course, such an assistant should include robust mechanisms for error handling and validation of its outputs, essential to ensure accuracy and to avoid or at least catch hallucinations (Smith et al., 2024), although on the other hand, at the fast pace these models evolve, one could expect such problems to decrease significantly in the near future.

## 6 PRACTICAL CHALLENGES AND THE PATH FORWARD

When tracing a roadmap for the future of software for immersive and interactive molecular graphics, some important considerations must be taken into account, including ergonomics, user fatigue, and VR sickness. All three are tightly linked to the evolution of headset technology and other broader, merely computational and technical hurdles.

Head‐mounted displays can feel heavy after a few minutes of use (Kim & Shin, 2021), a problem that will be alleviated as devices become lighter or are split into separate wearable parts—although the latter means more components such as wires and batteries hanging around, which makes the experience less seamless and less straightforward, as in the headsets available around a decade ago. Another problem that we can expect will lessen with newer generations of headsets is that of VR sickness, which notably also happens in AR relying on camera passthrough (Kaufeld et al., 2022). Another problem from the user side is posed by the ergonomic challenges of direct hand‐tracking, as mid‐air gestures can lead to significant arm fatigue (Chen & Wu, 2023; Khan et al., 2024). For the fine, prolonged manipulations common in molecular modeling, this physical strain could become a significant barrier. Future systems should incorporate solutions to mitigate this, such as mapping small, comfortable hand motions to larger virtual movements, allowing the joint use of hand gestures and controllers, and offloading complex commands to other modalities, most notably VUIs.

The vision of a fully interactive, multiuser “Holodeck” also rests on surmounting significant computational and networking challenges. Real‐time iMD is computationally expensive, both at the level of the calculation itself and of data transfer, and its scalability to large biomolecular complexes or materials systems remains a critical hurdle. Hybrid approaches, such as performing full simulations only on demand while using faster, approximate force fields for real‐time feedback, as in the HandMol prototype that only runs minimization when molecules are released, could provide a practical path forward.

Furthermore, for collaboration to be effective in a scientific context, platforms must ensure robust data synchronization, manage network latency to maintain immersion, and guarantee data security and reproducibility—all non‐trivial technical problems in multiuser tools.

## 7 CONCLUSIONS, AND A DRAFT DEVELOPMENT ROADMAP

The evolution of molecular graphics has equipped us with outstanding tools for visualization. Now, the imperative is to move toward truly interactive systems that prioritize manipulation, collaboration, and computer‐assisted work. Technology, especially (wearable) robotics, bioengineering, and of course the computer sciences, have given us tools that, already today, can radically transform how we do research and education, both in the wet and dry labs (Aspuru‐Guzik et al., 2018; Seifrid et al., 2022). As we have imagined it here, the future “Holodeck for molecular graphics and modeling” leverages accessible environments in which scientists can use intuitive hand gestures alongside natural language commands, receive rich multi‐sensory feedback, collaborate seamlessly with peers, and ground their interactions in real‐time physical calculations accessed via APIs or direct simulation (Figure 2).

If it can be realized, the shift envisioned here could unlock new levels of perception, intuition, and collaboration among humans and with computers, accelerating discovery, knowledge sharing, and learning. Realizing this vision requires a roadmap, laying out the incremental developments needed. The first step is refining today's proof‐of‐concept tools into robust platforms that address the key challenges of ergonomics, scalability, and security discussed throughout. The final goal is a seamless human–computer partnership that, in turn, facilitates human–human collaboration, all rigorously validated for reliability, intuitive design, and tangible impact on the speed and quality of scientific discovery and learning—the latter requiring well‐designed and rigorously analyzed benchmarks and user tests against established 2D interfaces, as discussed above. To contextualize the current landscape and the path toward the “Molecular Holodeck,” Table 1 provides a comparative summary of features across several existing platforms versus the fully realized, aspirational vision.

**TABLE 1:** Current state of the art, indicating limitations, and development aspirations for key features in immersive molecular modeling to achieve a fully realized “Molecular Holodeck.”

Feature	State of the art as of December 2025 (studies/tools/technology)	Aspirations, aiming at a “Molecular Holodeck”
Immersive visualization experience	Limited resolution in headsets, which additionally are heavy WebXR software on smartphones appears of better resolution; smartphones are lighter than consumer headsets Sickness in AR and VR experienced by some users in some circumstances	Graphics of higher resolution and fidelity, robust to large systems. Good blend with real world for AR, ideally not screen see‐through but superimposing image onto real‐world light, not only to improve the experience but also to reduce sickness Lightweight headsets, possibly glasses/spectacles‐like, for less fatigue
Direct hand manipulation	Apps support either controllers or hand tracking, but not mixed inputs, although this is technical feasible already today Hand moves in mid‐air can produce fatigue	Fully articulated, intuitive, and fatigue‐aware hand gestures Combine hand gestures with controllers to minimize fatigue and also to provide fine‐tuned operations via joystick, buttons, and so forth, or virtual equivalents
Multiuser Collaboration	Smooth experience dependent on bandwidth Co‐location API not available in all SDKs, preventing proper coordination of real and virtual spaces when users are physically close Issues with data security Issues with reproducibility of hand‐driven manipulations	Seamless, scalable, secure sessions accessible across multiple platforms Robust to bandwidth issues Robust to co‐located and remote collaboration (i.e., users in the same or distant physical spaces, respectively) Secure data transfer and communication; and ensure reproducibility
Real‐time Physics (iMD)	Few usable tools available (e.g., Narupa, NanoVer and the HandMol prototypes) For the moment, good performance is limited to few tens of thousands atoms	Scalable for large systems, possibly balancing on‐demand accuracy with real‐time feedback
Haptic feedback	No consumer technology allows this today; remains experimental only. Pseudo‐haptics offer potential	Providing rich physical sensations or other perception of steric clashes, forces, tensions, and so forth
Voice user interface (VUI)	Under slow development; so far only in the HandMol prototypes	Coupled to AI assistant (see next line)
AI intelligent assistant	Remains experimental; some basic functionalities in HandMol prototypes	Powered by LLM or reasoning agents, for data retrieval, analysis, problem detection, agent‐assisted and automated operations
Cross‐platform access	Out of the box only for WebXR‐based software; while some non‐web software is compiled for different platforms	Fully inclusive (XR, desktop, mobile access to same session)

Together with multimodal AI systems for molecular modeling and design, like those emerging for various chemical and biological application areas (Abriata, 2024; Batatia et al., 2025; Bozal‐Ginesta et al., 2025; Choi et al., 2025; Narayanan et al., 2025), a “Holodeck” in which a geographically dispersed team meets to collaboratively design a new inhibitor, protein, or material by discussing and making adjustments on the structures by applying their expertise at multiple scales and assisted by computer feedback to model physics and augmented human intuition with data is likely to be a reality within a decade.

## AUTHOR CONTRIBUTIONS

**Adrian J. Mulholland:** Conceptualization; investigation; writing – review and editing; methodology. **Luciano A. Abriata:** Conceptualization; investigation; funding acquisition; writing – original draft; writing – review and editing; visualization.

## Data Availability

Data sharing not applicable to this article as no datasets were generated or analysed during the current study.
